# Supplementary material for: Modeling glioblastoma heterogeneity as a dynamic network of cell states
Source: Mol Syst Biol. 2021 Sep 16;17(9):e10105. doi: 10.15252/msb.202010105 (PMC8444284; doi:10.15252/msb.202010105)
Supplement: Supplementary file 6 — Source Data for Figure 5 [file MSB-17-e10105-s004.zip › Figure5A_sourcedata/GSEA_3071/hallmarks_stateIII.GseaPreranked.1623742540963/HALLMARK_HYPOXIA.html]

Details for gene set HALLMARK\_HYPOXIA[GSEA]

|  || Dataset | state33071 |
| Phenotype | NoPhenotypeAvailable |
| Upregulated in class | na\_neg |
| GeneSet | HALLMARK\_HYPOXIA |
| Enrichment Score (ES) | -0.382045 |
| Normalized Enrichment Score (NES) | -2.2412527 |
| Nominal p-value | 0.0 |
| FDR q-value | 0.0012723891 |
| FWER p-Value | 0.009 |
Table: GSEA Results Summary

  

Fig 1: Enrichment plot: HALLMARK\_HYPOXIA      
 Profile of the Running ES Score & Positions of GeneSet Members on the Rank Ordered List

  

| PROBE | GENE SYMBOL | GENE\_TITLE | RANK IN GENE LIST | RANK METRIC SCORE | RUNNING ES | CORE ENRICHMENT || 1 | PHKG1 |  |  | 23 | 0.518 | 0.0093 | No |
| 2 | MT2A |  |  | 31 | 0.499 | 0.0221 | No |
| 3 | MIF |  |  | 68 | 0.445 | 0.0258 | No |
| 4 | ENO1 |  |  | 241 | 0.314 | -0.0096 | No |
| 5 | GAPDH |  |  | 320 | 0.286 | -0.0214 | No |
| 6 | HK2 |  |  | 432 | -0.250 | -0.0428 | No |
| 7 | ALDOA |  |  | 440 | -0.251 | -0.0373 | No |
| 8 | NDST2 |  |  | 447 | -0.251 | -0.0315 | No |
| 9 | ATF3 |  |  | 515 | -0.255 | -0.0414 | No |
| 10 | GPC4 |  |  | 593 | -0.259 | -0.0537 | No |
| 11 | MAFF |  |  | 735 | -0.266 | -0.0825 | No |
| 12 | GCNT2 |  |  | 1038 | -0.281 | -0.1526 | No |
| 13 | FOXO3 |  |  | 1088 | -0.284 | -0.1569 | No |
| 14 | GAA |  |  | 1151 | -0.287 | -0.1646 | No |
| 15 | PFKFB3 |  |  | 1519 | -0.307 | -0.2508 | No |
| 16 | KLF6 |  |  | 1596 | -0.312 | -0.2614 | No |
| 17 | ALDOC |  |  | 1613 | -0.313 | -0.2563 | No |
| 18 | CDKN1A |  |  | 1631 | -0.314 | -0.2515 | No |
| 19 | SELENBP1 |  |  | 1655 | -0.315 | -0.2482 | No |
| 20 | PDK1 |  |  | 1723 | -0.320 | -0.2562 | No |
| 21 | SRPX |  |  | 1747 | -0.322 | -0.2527 | No |
| 22 | DPYSL4 |  |  | 1761 | -0.322 | -0.2466 | No |
| 23 | ACKR3 |  |  | 1865 | -0.328 | -0.2637 | No |
| 24 | GYS1 |  |  | 1894 | -0.329 | -0.2613 | No |
| 25 | SLC2A1 |  |  | 1961 | -0.333 | -0.2686 | No |
| 26 | STBD1 |  |  | 1968 | -0.334 | -0.2604 | No |
| 27 | ZNF292 |  |  | 1992 | -0.335 | -0.2565 | No |
| 28 | DDIT4 |  |  | 2027 | -0.337 | -0.2554 | No |
| 29 | NDST1 |  |  | 2049 | -0.338 | -0.2510 | No |
| 30 | SLC2A3 |  |  | 2117 | -0.343 | -0.2583 | No |
| 31 | PLAUR |  |  | 2181 | -0.347 | -0.2644 | No |
| 32 | MXI1 |  |  | 2182 | -0.347 | -0.2542 | No |
| 33 | SLC37A4 |  |  | 2200 | -0.349 | -0.2484 | No |
| 34 | ENO2 |  |  | 2366 | -0.363 | -0.2805 | No |
| 35 | HMOX1 |  |  | 2415 | -0.366 | -0.2822 | No |
| 36 | SDC2 |  |  | 2537 | -0.376 | -0.3026 | No |
| 37 | STC2 |  |  | 2570 | -0.379 | -0.2997 | No |
| 38 | HOXB9 |  |  | 2651 | -0.386 | -0.3091 | No |
| 39 | TGM2 |  |  | 2933 | -0.413 | -0.3699 | Yes |
| 40 | JUN |  |  | 2935 | -0.413 | -0.3580 | Yes |
| 41 | SIAH2 |  |  | 2946 | -0.414 | -0.3485 | Yes |
| 42 | CSRP2 |  |  | 2974 | -0.417 | -0.3432 | Yes |
| 43 | SCARB1 |  |  | 2975 | -0.417 | -0.3310 | Yes |
| 44 | BHLHE40 |  |  | 2981 | -0.417 | -0.3200 | Yes |
| 45 | PAM |  |  | 3043 | -0.425 | -0.3234 | Yes |
| 46 | TIPARP |  |  | 3072 | -0.428 | -0.3181 | Yes |
| 47 | BCL2 |  |  | 3081 | -0.429 | -0.3076 | Yes |
| 48 | TNFAIP3 |  |  | 3088 | -0.430 | -0.2965 | Yes |
| 49 | P4HA2 |  |  | 3174 | -0.441 | -0.3056 | Yes |
| 50 | CHST3 |  |  | 3175 | -0.441 | -0.2926 | Yes |
| 51 | HSPA5 |  |  | 3193 | -0.442 | -0.2841 | Yes |
| 52 | WSB1 |  |  | 3218 | -0.445 | -0.2772 | Yes |
| 53 | SLC6A6 |  |  | 3270 | -0.454 | -0.2771 | Yes |
| 54 | ANKZF1 |  |  | 3302 | -0.460 | -0.2716 | Yes |
| 55 | NEDD4L |  |  | 3406 | -0.481 | -0.2842 | Yes |
| 56 | EGFR |  |  | 3433 | -0.488 | -0.2766 | Yes |
| 57 | TPST2 |  |  | 3437 | -0.489 | -0.2630 | Yes |
| 58 | SERPINE1 |  |  | 3474 | -0.499 | -0.2577 | Yes |
| 59 | HEXA |  |  | 3537 | -0.523 | -0.2584 | Yes |
| 60 | FOS |  |  | 3621 | -0.561 | -0.2635 | Yes |
| 61 | TPBG |  |  | 3638 | -0.570 | -0.2509 | Yes |
| 62 | CA12 |  |  | 3694 | -0.611 | -0.2472 | Yes |
| 63 | KLHL24 |  |  | 3697 | -0.613 | -0.2297 | Yes |
| 64 | RORA |  |  | 3716 | -0.630 | -0.2159 | Yes |
| 65 | DCN |  |  | 3723 | -0.633 | -0.1988 | Yes |
| 66 | AKAP12 |  |  | 3740 | -0.660 | -0.1836 | Yes |
| 67 | F3 |  |  | 3764 | -0.693 | -0.1692 | Yes |
| 68 | STC1 |  |  | 3770 | -0.702 | -0.1499 | Yes |
| 69 | TGFBI |  |  | 3781 | -0.713 | -0.1315 | Yes |
| 70 | MAP3K1 |  |  | 3796 | -0.737 | -0.1135 | Yes |
| 71 | CXCR4 |  |  | 3802 | -0.750 | -0.0928 | Yes |
| 72 | PDGFB |  |  | 3803 | -0.751 | -0.0707 | Yes |
| 73 | BGN |  |  | 3812 | -0.761 | -0.0504 | Yes |
| 74 | NCAN |  |  | 3813 | -0.761 | -0.0281 | Yes |
| 75 | COL5A1 |  |  | 3815 | -0.764 | -0.0059 | Yes |
| 76 | IGFBP3 |  |  | 3903 | -1.199 | 0.0067 | Yes |
Table: GSEA details [plain text format]

  

Fig 2: HALLMARK\_HYPOXIA: Random ES distribution      
 Gene set null distribution of ES for **HALLMARK\_HYPOXIA**
